# Improving patient flow: ethnicity and socio-economic status associated with delayed discharge from hospital in patients with COVID-19 infection: An observational epidemiological study

**DOI:** 10.1177/17449871241310384

**Published:** 2025-08-29

**Authors:** Colin J Crooks, Joe West, Tasso Gazis, Joanne R Morling, Mark Simmonds, Irene Juurlink, Steve Briggs, Simon Cruickshank, Susan Hammond-Pears, Dominick Shaw, Timothy R Card, Andrew Fogarty

**Affiliations:** Associate Professor in Clinical Epidemiology, Nottingham Digestive Diseases Centre, School of Medicine, University of Nottingham, Nottingham, UK; NIHR Nottingham Biomedical Research Centre (BRC), Nottingham University Hospitals NHS Trust and the University of Nottingham, Nottingham, UK; Nottingham University Hospitals NHS Trust, Nottingham, UK; Professor of Epidemiology, Lifespan and Population Health, School of Medicine, University of Nottingham, Nottingham, UK; NIHR Nottingham Biomedical Research Centre (BRC), Nottingham University Hospitals NHS Trust and the University of Nottingham, Nottingham, UK; Nottingham University Hospitals NHS Trust, Nottingham, UK; East Midlands Academic Health Science Network, University of Nottingham, Nottingham, Nottingham, UK; Clinical Director for Urgent and Emergency Care, Nottingham University Hospitals NHS Trust, Nottingham, UK; MRC Clinician Scientist in Public Health, Lifespan and Population Health, School of Medicine, University of Nottingham, Nottingham, UK; Divisional Director, Medicine, Nottingham University Hospitals NHS Trust, Nottingham, UK; Enterprise Data Architect, Nottingham University Hospitals NHS Trust, Nottingham, UK; Advanced Analyst, Nottingham University Hospitals NHS Trust, Nottingham, UK; Principal Analyst, Nottingham University Hospitals NHS Trust, Nottingham, UK; Improvement Lead/Advanced Analyst, Nottingham University Hospitals NHS Trust, Nottingham, UK; East Midlands Academic Health Science Network, University of Nottingham, Nottingham, Nottingham, UK; Professor of Respiratory Medicine, Respiratory Biomedical Research Centre, University of Leicester, Leicester, UK; Associate Professor in Clinical Epidemiology, Lifespan and Population Health, School of Medicine, University of Nottingham, Nottingham, UK; NIHR Nottingham Biomedical Research Centre (BRC), Nottingham University Hospitals NHS Trust and the University of Nottingham, Nottingham, UK; Nottingham University Hospitals NHS Trust, Nottingham, UK; Reader in Clinical Epidemiology, Lifespan and Population Health, School of Medicine, University of Nottingham, Nottingham, UK; NIHR Nottingham Biomedical Research Centre (BRC), Nottingham University Hospitals NHS Trust and the University of Nottingham, Nottingham, UK; Nottingham University Hospitals NHS Trust, Nottingham, UK

**Keywords:** age, comorbidities, ethnicity, hospital discharge, in-patients, socio-economic class

## Abstract

**Background:**

Understanding the reasons for delays in leaving hospital once an in-patient is considered ready for discharge is important to inform the development of interventions to improve patient flow through resource-stressed healthcare systems.

**Aims:**

To identify risk factors for delayed discharge from hospital during the COVID-19 pandemic.

**Methods:**

The study population was all patients admitted with COVID-19 infection from February 2020 to September 2021 to a large UK teaching hospital.

**Results:**

Data were available from 7929 admission events with a median delay of 0.20 days from being considered medically safe for discharge and the discharge date. Age older than 60 years (+2.23 days), White ethnicity (+1.58 days compared to SE Asian), living in an area of increased affluence (+0.13 days per decile decrease in deprivation) and having two or more comorbidities (+1.82 days; compared to no comorbidities) were associated with delayed discharge.

There was a total potential saving of over 22,000 bed-days if all patients had been discharged when they were considered medically safe.

**Conclusions:**

Early identification of patients at an increased risk of a delayed discharge may allow development of appropriate anticipatory interventions, and inform policymakers to help identify and minimise bottlenecks at the institutional level.

## Introduction

Healthcare systems in the 21st century have to deliver high-quality medical care to an aging population. Factors which prolong hospital stays after the patient is medically safe for discharge constitute a public heath area of interest, particularly in England, as the bed occupancy rate is very high at approximately 84% overall, with 82 Trusts exceeding the 85% rate which is considered the limit for safe and efficient delivery of healthcare. Thirty-five of these Trusts are having bed occupancy rates higher than 90% (O’Dowd, 2021). A recent scoping review has identified that there remain significant gaps and limitations in the evidence base required to understand delayed discharges from hospital (Cadel et al., 2021). In addition, the situation is relatively dynamic, with particular issues with high demand for beds within an inelastic system with limited reserve capacity in winter when demand for medical beds increases. It is likely that these pressures will also impact on other healthcare systems other than in England, although the variety of differing healthcare models for both funding and delivering healthcare make international comparisons challenging.

The responsibility of managing delayed discharges from hospital adds to the workload on ward nursing staff, in addition to their more acute clinical responsibilities. It also negatively impacts on the health and social care management teams, who are obliged to manage finite healthcare resources to handle high patient numbers, often to the detriment of delivery of healthcare elsewhere.

The concept of electronic medical records is well established in the United Kingdom and was initially introduced to primary care over two decades ago (McMillan et al., 2018). Adoption of electronic medical records into secondary care has proven more challenging, and replacing traditional paper medical records has proven an incremental process that is ongoing. This has led to a hybrid model in some hospitals where simple physiological measurements such as clinical observations are collected and uploaded to a central database using smartphones and electronic tablet interfaces, and other clinical variables are also appended to the same dataset. The ultimate aim is for all clinical observations and records to be electronically stored, and paper records to be eliminated from secondary care settings (Parkin, 2016).

This introduction of electronic medical records systems into hospitals aims to improve data collection in real-time and utilise this to improve patient care and manage patient flow. One of the factors that has been added to the electronic dataset is a ‘Medically Safe For Discharge’ status for each patient, which is reviewed daily. This has been designed to help understand the status of each patient in the hospital and give an overview of the requirement for help in facilitating safe hospital discharges. However, it also allows an opportunity to better understand the factors that modify patient flow from admission to discharge, and hence may help design interventions to improve this over time. The introduction of a ‘Medically Safe for Discharge’ label for each patient is a relatively novel concept, and this approach enables the analysis of large numbers of patients relatively efficiently, without the need for manual data collection.

The COVID-19 pandemic resulted in a natural experiment, whereby large numbers of patients have been admitted to hospitals with the same infection. This allowed the exploration of factors that may result in delayed discharge from hospital in patients who were deemed medically safe for discharge, and who all had the same infection. This analysis explored how age, sex and ethnic group may modify the time to discharge in patients who were admitted with COVID-19 infection and were deemed medically safe for discharge.

The research questions of interest were:

- What are the associations of age, sex, ethnic group, socio-economic status and the presence of comorbidities on delays in leaving hospital for in-patients who had been labelled ‘Medically Safe for Discharge’.

## Methods

We conducted an observational epidemiological study using routinely collected electronic data for patients admitted to Nottingham University Hospitals NHS Trust between 1 February 2020 and 30 September 2021 within 60 days of a confirmed SARS-CoV-2 diagnosis. This is a large teaching hospital in the United Kingdom which admits all unselected patients within its catchment area. The data only allow identification of patients with a diagnosis of COVID-19 infection and cannot determine if it was the primary reason for admission, hospital-acquired or sub-clinical. Data were available for the date at which these patients were labelled as, ‘Medically Safe for Discharge’, and also the date when they left the hospital. The data thus included patients who had received a ‘Medically Safe for Discharge’ label and then remained in hospital for a period of time afterwards, and patients who were discharged quickly without formally receiving a ‘Medically Safe for Discharge’ label. A medically safe for discharge decision was made on a daily basis by a senior decision-maker on each ward. This allowed the excess length of stay to be calculated as non-medical concerns were addressed. A sensitivity analysis restricted to only patients who had received a label of a medically safe for discharge before the discharge date in their records was performed as a secondary analysis as these were the patients who experienced a delay in leaving hospital and hence are of particular interest.

Median and interquartile range for excess length of stay were stratified by sex, socio-economic status by indices of multiple deprivation (IMD) decile, age at admission categories, a simple measure of concurrent comorbidities as defined by the Charlson index (Glasheen et al., 2019) and recorded ethnicity of White, Mixed, Indian, Pakistani, Black and other or unknown ethnic group. These were further grouped as White, Indian/Pakistani, Black/mixed and other/unknown to account for small numbers. Decile of the ranking of a patient’s residence by the IMD for England was used, with those areas at highest risk of deprivation ranked in the 1st (1–10%) decile, and the lowest risk of deprivation in the 10th (91–100%) decile. Patients not mapped to a postcode and therefore for whom an IMD decile was not available were excluded from the analysis. We fitted a multivariate model to assess whether the variations in length of stay within different ethnicity and deprivation strata were independent of each other, and whether they were explained by differences in age or sex. We modelled the excess length of stay as a continuous linear outcome, so that fitted coefficients represent the increase in excess stay in days that was associated with a unit increase in the respective covariate. We included age, sex, ethnic group, socio-economic IMD decile and Charlson comorbidity score as explanatory variables. Socio-economic IMD decile was included as a continuous variable and tested for a departure from linear trend with a likelihood ratio test. A random intercept at patient level was included to model the correlation between multiple admissions. All analyses were performed using version 4.1.1 of the R programming language (The R Project for Statistical Computing).

### Ethical approval

Approval for this work was granted via an Nottingham University Hospitals’ NHS Trust Clinical Effectiveness Team audit (reference: 21-294C). The analysis used anonymised patient data, and no individual patient consent was required.

## Results

Data were available from 7929 admissions with a delay in discharge from 5784 patients who were labelled ‘Medically Safe for Discharge’, with a median age of 66 years (interquartile range (IQR): 46 to 80 years). There was an overall median delay between a patient having a medically safe for discharge date recorded and time of discharge of +0.19 days (IQR: +0.09 to +1.20). The study population is described in Table 1, along with the length of stay after the patient was labelled ‘Medically Safe for Discharge’.

**Table 1.** Summary statistics of univariate associations between length of stay after patient deemed medically safe for discharge and sex, age and ethnic group in patients with a COVID-19 flagged admission within 60 days of first COVID-19 infection.

Exposure	All patients (assuming patients were medically safe on day of discharge if not flagged medically safe prior to discharge)			Patients who were flagged medically safe before discharge		
	Number of patient admissions	Median number of days to discharge once medically safe	Interquartile range	Number of patient admissions	Median number of days to discharge once medically safe	Interquartile range
Total patients	5784	0.20	(0.10–1.32)	2556	2.04	(0.29–7.54)
Total admissions*	7929	0.19	(0.09–1.20)	3359	1.99	(0.28–7.20)
Sex						
Male	3850	0.19	(0.09–1.06)	1624	1.39	(0.26–7.19)
Female	4079	0.20	(0.09–1.37)	1735	2.10	(0.30–7.23)
Age category						
⩽60 years	3499	0.13	(0.06–0.24)	736	0.31	(0.14–2.15)
>60 years	4430	0.32	(0.13–4.09)	2623	2.39	(0.40–8.13)
Ethnicity recorded						
White	5377	0.22	(0.10–2.01)	2513	2.10	(0.31–7.35)
Other or Unrecorded	1607	0.17	(0.08–0.48)	593	1.39	(0.24–7.05)
Indian/Pakistani	586	0.13	(0.06–0.25)	137	0.32	(0.17–2.36)
Black/Mixed	359	0.17	(0.08–0.31)	116	0.53	(0.18–4.06)
Indices of multiple deprivation						
1–10% (Highest deprivation risk decile)	1334	0.17	(0.08–0.46)	501	1.22	(0.21–6.28)
11–20%	1220	0.18	(0.09–0.98)	486	1.31	(0.25–5.27)
21–30%	769	0.18	(0.09–0.97)	306	1.38	(0.25–6.62)
31–40%	762	0.18	(0.08–0.52)	281	1.30	(0.26–8.13)
41–50%	615	0.19	(0.10–1.84)	276	2.18	(0.30–7.95)
51–60%	615	0.21	(0.09–1.32)	254	2.06	(0.33–7.33)
61–70%	620	0.22	(0.09–2.04)	293	2.08	(0.35–7.23)
71–80%	525	0.21	(0.09–1.22)	238	1.32	(0.30–6.96)
81–90%	675	0.22	(0.11–2.16)	316	2.32	(0.40–9.39)
91–100% (Lowest deprivation risk decile)	794	0.25	(0.11–3.01)	408	2.40	(0.31–7.54)
Charlson index of comorbidities						
0	2550	0.15	(0.07–0.32)	715	1.14	(0.21–6.16)
1	1689	0.18	(0.09–0.98)	706	1.31	(0.25–6.24)
⩾2	3690	0.25	(0.11–2.50)	1938	2.21	(0.36–8.03)

* Note: The number of admissions is higher than the number of patients due to readmissions of the same patient.

The final analysis adjusting for all demographic factors is presented in Table 2 both for the total population and the sensitivity analysis restricted to patients with a ‘Medically Safe for Discharge’ label prior to the admission date. For the main analysis of all admissions, increasing age was associated with an increased length of stay after the patient was deemed medically safe for discharge, with those aged over 60 years remaining in hospital for +2.23 (95% confidence interval (CI): +1.53 to +2.92) days longer than those aged 60 years and younger when adjusted for all other covariates. A comparison with age categorised into < 51 years, 51–60 years, 61–70 years, 71–80 years and >80 years had minimal effect on the coefficients for ethnicity and deprivation (<0.1 day), so the simpler model is presented in Table 2. Individuals who were of Indian or Pakistani ethnicity had a shorter stay in hospital once considered medically safe for discharge, staying for −1.58 (95% CI: −2.86 to −0.30) days less than individuals of White ethnicity. Decreasing multiple deprivation (or more affluence) was associated with longer length of stay in hospital once the patient was medically safe for discharge, with a one decile decrease in deprivation (or more affluence) ranking being associated with a +0.13 days (95% CI: +0.02 to +0.23) increase in length of stay once the patient was medically stable. A test for a departure from linear trend for deprivation was not significant (*p* = 0.3687). Finally, those individuals with more than one morbidity as coded by the Charlson index had a longer length of stay once medically treated than individuals with no comorbidities (+1.82 days; 95% CI: +1.06 to +2.59). Sex was not associated with delayed stay in hospital once the patient was considered safe for discharge in the main analysis, with CIs that included the null (−0.20 days (−0.82 to +0.43) for females compared to males).

**Table 2.** Multivariate analysis of association between length of stay after patient deemed medically safe for discharge and sex, age, socio-economic status and ethnic group.

Exposure	Days to discharge once medically safe Coefficient (95% confidence interval)	
	All patients (assuming patients were medically safe on day of discharge if not flagged medically safe prior to discharge)	Patients who were flagged medically safe before discharge
Male	0 (reference)	0 (reference)
Female	−0.20 (−0.82 to +0.43)	−0.98 (−2.21 to +0.26)
⩽60 years	0 (reference)	0 (reference)
>60 years	+2.23 (+1.53 to +2.92)	+1.07 (−0.50 to +2.63)
White	0 (reference)	0 (reference)
Indian and Pakistani	−1.58 (−2.86 to −0.30)	−2.93 (−6.07 to +0.20)
Black and mixed	−0.75 (−2.26 to +0.75)	−1.86 (−5.29 to +1.57)
Other or unrecorded	−0.75 (−1.53 to +0.03)	−1.16 (−2.78 to +0.46)
Indices of multiple deprivation ranking of residence by decile (Highest deprivation risk = 1, lowest deprivation risk = 10. Covariate therefore indicates increase in discharge delay with each decile reduction in deprivation risk)	+0.13 (+0.02 to +0.23)	+0.25 (+0.05 to +0.45)
Charlson index – No morbidity	0 (reference)	0 (reference)
Charlson index – Single morbidity	+0.03 (−0.81 to +0.87)	−0.40 (−2.25 to +1.45)
Charlson index – Multiple or severe morbidity	+1.82 (+1.06 to +2.59)	+1.71 (+0.13 to +3.28)
Observations (patient admissions)	7929	3359

Multi-level model with random effect included for patient.

There was a total potential saving of over 22,000 bed-days if all patients had been discharged when they were considered medically safe to leave the hospital.

Similar, but generally less precise associations were observed in the sensitivity analysis restricted to the subset of patients who had a ‘Medically Safe for Discharge’ label prior to the date for discharge.

## Discussion

This is the first use of an electronic ‘Medically Safe for Discharge’ label to explore potential factors that delay discharge from hospital once the patient has been considered as medically safe for discharge in a large population of patients from a single centre with the same disease process. The data demonstrate that increasing age, a White ethnic group, coming from a more affluent area of residence and having two or more comorbidities are associated with a longer duration in hospital once labelled medically safe for discharge. These data are important and illustrate the scale of the problem in a population who were treated for COVID-19 infection over a 20-month period in a busy UK teaching hospital. As such it should be considered a ‘proof-of-concept’ study that demonstrates the potential utility of a simple modification to the electronic nursing records to understand healthcare delivery at the institutional level.

Increasing age and increasing comorbidities were both risk factors for a prolonged stay in hospital after being labelled ‘Medically Safe for Discharge’. This is consistent with the current understanding of the topic (The Health Foundation, 2023; Walsh, 2021), and as the UK population demography becomes older (Barton et al, 2024), with more people living with comorbidities (National Institute of Health Research, 2021), may have implications for preparations to provide healthcare for future generations in the country. It is important to emphasise that these effects were from a mutually adjusted model; hence, age is a risk factor for prolonged stay in hospital after adjustment for comorbidities. This may reflect the absence of supportive networks that can arise when living alone, possibly after the loss of a companion, as well as the frailty that can accompany the ageing process.

The (i) markedly shorter length of stay for individuals from Indian/Pakistani backgrounds, and (ii) that coming from a more affluent background was associated with a longer length of stay once medically safe are new observations. They will require further investigation to understand the mechanisms underlying them and if they are present in diseases other than COVID-19 infection. This is possible that patients from these ethnic groups often have supportive families who live locally.

## Strengths and limitations of the study

The strengths of these data are that they come from a complete cohort of all patients admitted to a single UK teaching hospital with COVID-19 infection and use routinely collected data for the analysis. These data are all collected electronically and hence represent a complete dataset with no missing values to facilitate an optimal analysis. This is the first time that an electronic label of ‘Medically Safe for Discharge’ has been used as prior studies have used retrospective case note reviews in older patients (Challis et al., 2014; Moore et al., 2018; Victor et al., 2000).

One limitation of using real-world health data outside a controlled experimental environment is that the threshold for healthcare professional deciding when the patient is clinically safe for discharge is not standardised, but may vary. However, this makes the data reflective of the reality of a healthcare system and does not detract from the associations observed. The real-world nature of these data also demonstrates that patients were readmitted after initial discharge from hospital, but it is not possible to categorise the cause of these readmissions. Our experience from working through the pandemic suggests that they will have a variety of causes, ranging from complications of the initial COVID-19 infection to difficulty managing at home when other members of the family may also be unwell with COVID-19 infection. As these data were collected during a viral pandemic, caution should be taken when generalising them to other times, and we anticipate further studies from outside the viral pandemic time period will be required to clarify this issue. The data were coded for ethnicity on arrival to hospital, and this is one area where measurement error may occur. The likelihood is that if there was uncertainty in the ethnic group, this would have been categorised as ‘other or unrecorded’, so patients in this category probably represent a very heterogenous population from an ethnic perspective.

A further limitation of this analysis is that the data do not provide detailed information on the factors that may delay the discharge from hospital. The application of a ‘Medically Safe for Discharge’ label is a very general one that provides limited information on individual barriers to leaving hospital, which will vary from patient to patient. These may include social support at home from family and professional carers as well as waiting for alternative residential locations with adequate nursing and social support (Nuffield Trust, 2024). Future studies on this topic may consider these factors, which may also increase understanding of the associations reported in this analysis. One possibility could include expanding the current binary ‘Medically Safe for Discharge’ label to one which provides more information on the reasons for the delayed discharges.

Our work builds on that of others researchers; a mixed-studies systematic review of the literature on studies of delayed discharge from hospital observed that delayed discharge was associated with mortality, infections, depression and reductions in patients’ mobility. However, they noted the ‘poor quality of the majority of the research means that implications for practice should be cautiously made’ (Rojas-García et al., 2018). Most studies on delayed discharge have been retrospective case note reviews in older patients (Abdelhalim et al., 2024). As a consequence, the factors noted to be associated with delayed discharge from hospital have been specific to this population and differ to our data and analysis. These have included cognitive impairment (Challis et al., 2014), admission to a care home (Challis et al., 2014; Moore et al., 2018; Victor et al., 2000) and the absence of a family carer (Moore et al., 2018; Victor et al., 2000).

These data are novel and have the potential to help start to understand the complex factors that are involved in facilitating and impeding discharge from hospital. The use of a population defined by a single disease process provides a degree of standardisation that makes any demographic or sociological factors that modify discharge from hospital a true association as opposed to secondary to confounding. The Nuffield Trust (The Nuffield Trust, (2024)) has highlighted the scale of problem of delayed discharge from hospitals in the United Kingdom, but these data allowed analysis in terms of the size of the issue across many hospitals in the United Kingdom, as opposed to at the level of the individual patient with an electronic time-stamp of when the patient was considered medically safe for discharge from hospital.

## Conclusions

To the best of our knowledge, this is the first observational study to use electronic data to investigate the factors that modify timing of discharge of patients from a hospital. As such, it sets the scene and highlights the need for further work to corroborate our findings in other large datasets in different healthcare settings. These data identify that increasing age, increasing number of morbidities and increased affluence are associated with a longer length of stay in hospital after being labelled ‘Medically Safe for Discharge’. Individuals from an Indian or Pakistani background have a shorter length of stay when medically safe than those from a White background.

Using electronic data to identify factors for delayed discharge has important implications for both nursing staff on the wards and also health and social policymakers who are responsible for making healthcare institutions efficient and functional. Developing patient flow pathways for individual hospitals will help identify local bottlenecks and hence inform the design and evaluation of interventions earlier in the admission timeline to try and minimise these issues (Cadel et al., 2021), hence improving the ability of hospitals to deliver optimal healthcare to their local populations. Introducing a ‘Medically Safe for Discharge’ label for hospital inpatients is relatively simple once the electronic medical records are established, and thus represents a small modification of current processes that has the potential to provide a useful tool for all individuals interested in understanding the complex factors that impede their efficient discharge from hospitals.

**Key points for policy, practice and further research**

- Patients who were older, of White ethnicity, living in more affluent areas and with two of more comorbidities had an increased risk of delayed discharge from hospital.
- In this group of patients with COVID-19 infection, this represented a total of over 22,000 extra bed-days over a 19-month period, compared to if these patients had been able to go leave hospital when considered medically safe for discharge.
- The relatively novel approach of using electronic medically safe for discharge labels may allow nursing staff in hospitals to identify patients at increased risk of a delayed discharge earlier, and thus develop appropriate anticipatory interventions.
